# Supplementary material for: Enhanced Autophagy in Damaged Laminar Tissue of Acute Laminitis Induced by Oligofructose Overloading in Dairy Cows
Source: Animals (Basel). 2023 Jul 31;13(15):2478. doi: 10.3390/ani13152478 (PMC10416948; doi:10.3390/ani13152478)

Supplementary Materials Figures (S1-S3)

Western blots Images Data (LC3II, Becilin1 and P62 Proteins)

Figure S1: Beclin-1

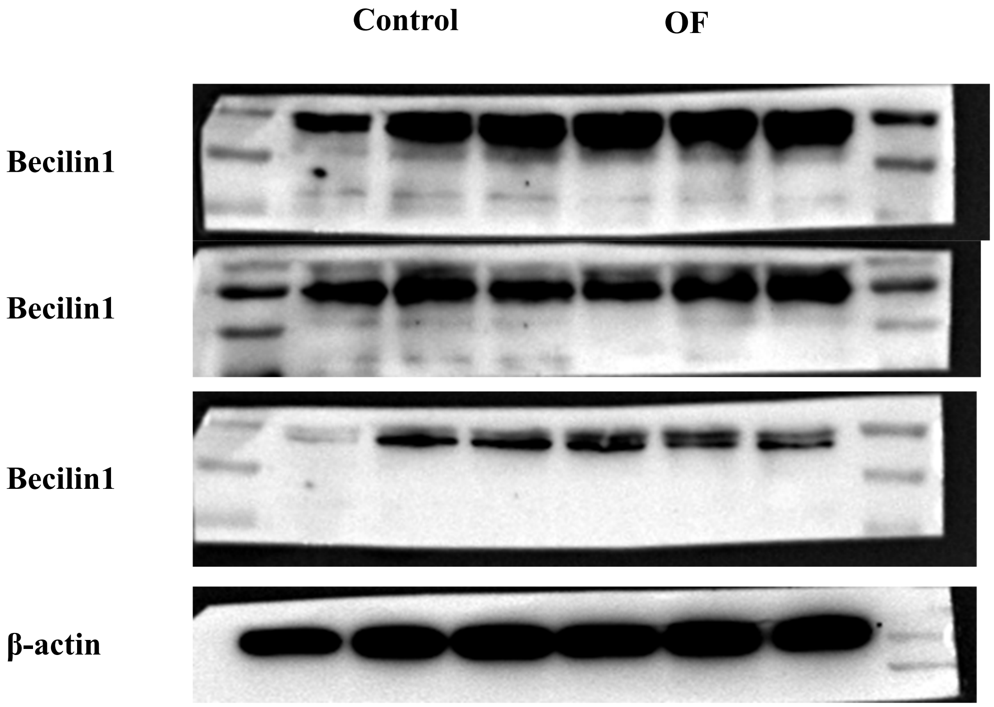

Figure S2:P62

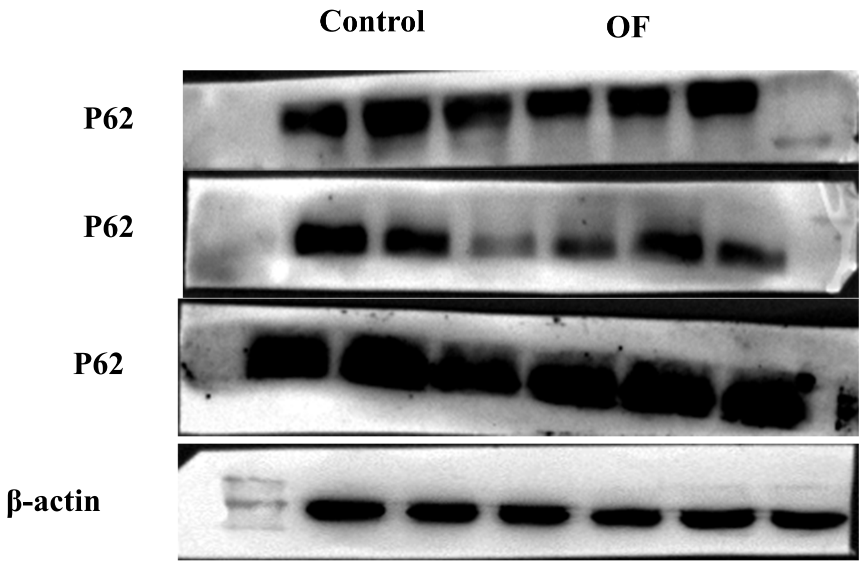

Figure S3:LC3II

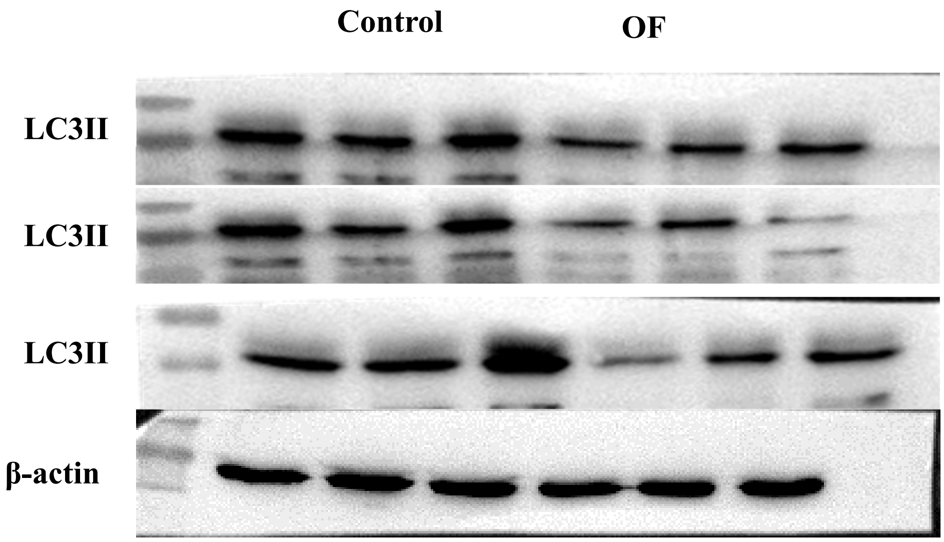

Supplement: Supplementary file 1 [file animals-13-02478-s001.zip › animals-2493363-supplementary.pdf]
